# Comparison of Tracer Kinetic Models for 68Ga-PSMA-11 PET in Intermediate Risk Primary Prostate Cancer Patients

**DOI:** 10.21203/rs.3.rs-3420161/v1

**Published:** 2023-11-01

**Authors:** Nathaniel J. Smith, Mark A. Green, Clinton D. Bahler, Mark Tann, Wendy Territo, Anne M. Smith, Gary D. Hutchins

**Affiliations:** Indiana University School of Medicine; Indiana University School of Medicine; Indiana University School of Medicine; Indiana University School of Medicine; Indiana University School of Medicine; Siemens Medical Solutions USA Inc: Siemens Healthcare USA; Indiana University School of Medicine

**Keywords:** 68Ga-PSMA-11 PET, tracer kinetic model, compartmental model, graphical model, Patlak analysis, primary prostate cancer, dynamic imaging

## Abstract

**BACKGROUND:**

^68^Ga-PSMA-11 positron emission tomography enables the detection of primary, recurrent, and metastatic prostate cancer. Regional radiopharmaceutical uptake is generally evaluated in static images and quantified as standard uptake values (SUV) for clinical decision-making. However, analysis of dynamic images characterizing both tracer uptake and pharmacokinetics may offer added insights into the underlying tissue pathophysiology. This study was undertaken to evaluate the suitability of various kinetic models for ^68^Ga-PSMA-11 PET analysis. Twenty-three lesions in 18 patients were included in a retrospective kinetic evaluation of 55-minute dynamic ^68^Ga-PSMA-11 pre-prostatectomy PET scans from patients with biopsy-demonstrated intermediate to high-risk prostate cancer. A reversible one-tissue compartment model, irreversible two-tissue compartment model, and a reversible two-tissue compartment model were evaluated for their goodness-of-fit to lesion and normal reference prostate time-activity curves. Kinetic parameters obtained through graphical analysis and tracer kinetic modeling techniques were compared for reference prostate tissue and lesion regions of interest.

**RESULTS:**

Supported by goodness-of-fit and information loss criteria, the irreversible two-tissue compartment model was selected as optimally fitting the time-activity curves. Lesions exhibited significant differences in kinetic rate constants (K_1_, k_2_, k_3_, Ki) and semiquantitative measures (SUV) when compared with reference prostatic tissue. The two-tissue irreversible tracer kinetic model was consistently appropriate across prostatic zones.

**CONCLUSIONS:**

An irreversible tracer kinetic model is appropriate for dynamic analysis of ^68^Ga-PSMA-11 PET images. Kinetic parameters estimated by Patlak graphical analysis or full compartmental analysis can distinguish tumor from normal prostate tissue.

## Background

Prostate cancer has an estimated lifetime incidence of 1 in every 9 men, but it is estimated that between 20% and 40% of prostate cancer diagnoses are unnecessary and attributable to widespread serum prostate-specific antigen testing [1, 2]. Surgery and radiotherapy significantly reduce the prevalence of metastatic disease progression, but may also cause erectile dysfunction and/or urinary incontinence [3]. Appropriately specific diagnostics can reduce the incidence of overtreatment and improve patient-specific outcomes. Positron emission tomography (PET) imaging with the urea-based prostate-specific membrane antigen (PSMA) targeted ^68^Ga-Glu-NH-CO-Lys-(Ahx)-HBED-CC (^68^Ga-PSMA-11) has greatly improved the diagnosis and treatment planning for prostate cancer, as upregulated PSMA expression has been linked with aggressive or advanced disease[4, 5].

The ^68^Ga-PSMA-11 tracer standardized uptake value (SUV) correlates with pathological Gleason grade and can support surgical planning as well as detect nodal metastases and biochemical recurrence[6, 7]. SUVs are commonly favored for their ease of clinical implementation, but SUVs depend on accurate dose and scanner cross-calibration, the time between injection and imaging, image acquisition characteristics (scanner, scatter/attenuation correction, reconstruction, frame duration), patient weight and radiopharmaceutical distribution characteristics, and may be affected by patient motion or partial volume effects. Therefore, differences in acquisition can make the comparison of SUVs across different patients and acquisition timepoints error-prone, especially when numerical cutoffs are used [8].

Kinetic modeling of tracer binding interactions reduces the impact of errors associated with patient weight, uptake timing, and dose calibration [9]. Unlike SUV-centered analysis and simple static images, dynamic PET imaging with ^68^Ga-PSMA-11 may be used to distinguish physiologic differences in receptor-ligand affinity, receptor availability, and ligand delivery and extraction, which are considered in aggregate with SUV analysis[10]. These physiologic parameters provide additional information which can improve tissue characterization [11, 12]. However, few studies have compared compartmental models for ^68^Ga-PSMA-11, and there is not a clear consensus for whether a reversible or irreversible two-tissue compartmental model optimally suits ^68^Ga-PSMA-11 PET data [13, 14].

^68^Ga-PSMA-11 is rapidly cleared from the blood, and blood metabolite components may be assumed negligible for the compartmental model [15]. This study aimed to verify the findings by Ringheim et al., and confirm the use of an irreversible two-tissue compartment model for ^68^Ga-PSMA-11 PET analysis [14].

## Methods

### Patients

Eighteen men with a total of 23 lesions were included in this retrospective evaluation (NCT04936334), after two patients were removed from the study cohort due to excessive motion during imaging. This study was approved by the institutional review board, and informed consent was obtained for all individuals prior to imaging. Men with histologically-proven prostate cancer before scheduled prostatectomy were eligible for this study if they were over the age of 18 and had at least NCCN intermediate risk disease or 3 cores of at least Gleason 3 + 4 disease. Patients needed to be able to lay still for the entire 60-minute PET/CT scan, and were excluded if they had received treatments with ionizing radiation within the past 30 days. The study patients had elevated PSA values (median 6.8, range 4.1–20.6) and enlarged prostates (median 40.4mL, range 27.3–89.4mL), and were primarily white (17/18). The median patient age was 65 (range: 52–75) and the median patient body weight was 90.7kg (range: 63.5–132.0 kg). A more complete charting of patient demographics is contained in Table 1.

### PET/CT Acquisition Protocol

Patients received a 55-minute dynamic PET acquired in list mode, centered over the pelvis. Images were acquired with a Siemens Biograph Vision 600 Edge scanner (Siemens Healthineers, Knoxville, USA). The ^68^Ga-PSMA-11 radiopharmaceutical was prepared as previously reported in the literature[16, 17]. At the start of the PET scan, patients received a bolus injection of [^68^Ga]-PSMA-11 (median 183.5 MBq, range 170.6–186.1 MBq), followed by a 10mL saline flush. PET images were reconstructed with a 3D ordered-subsets expectation maximization (OSEM) algorithm with point-spread function (PSF) and time of flight (TOF) [3i5s, 3.5mm FWHM spatial resolution, 210ps temporal resolution, 1.42 × 1.42 × 3.0mm voxels, 5mm Gaussian smoothing]. The images were corrected for decay, attenuation, scatter, dead time, random coincidences, and were detector-normalized. The PET images were then processed into 40 temporal frames (12 × 5s, 12 × 10s, 6 × 20s, 10 × 300s).

Computed tomography (CT) images were acquired sequentially with the PET scan (120 kV peak, 330ms exposure time, 658 mA tube current, 0.98 × 0.98 × 1.00mm voxels, 500mm field of view) using a soft tissue kernel (Br38).

### Image Analysis

The reconstructed PET/CT images were analyzed by a board-certified nuclear medicine physician and a board-certified urologist using in-house software (*Q-Image*) built using IDL (L3Harris Geospatial, Boulder, CO, USA). Forty cubic millimeter (~ 50 voxel) spherical reference regions of interest (ROIs) were sampled in the central, peripheral, and transitional prostatic zones in the left and right hemispheres. Separate ROIs were also contoured under physician guidance for the index lesion, contralateral reference region, and secondary lesions when present. Time-activity curves (TACs) were extracted in Bq/mL units. SUVs were calculated using the final 15 minutes of the scan, and mean SUV was calculated for each ROI.

### Image-derived Input Function

The image-derived input function (IDIF) was calculated using in-house software built in IDL. A spherical ROI (10.0 mm diameter) was placed on a linear segment of the iliac artery on a bolus phase PET image (approximately the first 60 seconds of data acquisition). Profiles across the vessel were generated at each location along the length of the vessel that fell within the boundaries of the localization spherical ROI. Each profile was fit with a vessel profile model (vessel width step function convolved with scanner resolution kernel) to estimate the vessel diameters. Vessel diameter estimates were then used to generate a 3D vessel model with a uniform background region that was large enough to capture all signal spillover into the vessel region. Simulated PET images generated by convolving the 3D vessel and background model were generated to estimate resolution distortion correction factors. Resolution distortion corrected time-activity curves for the vessel region were then generated using the distortion correction factors and original time-activity curves for the vessel and background regions. The distribution of ^68^Ga-PSMA-11 was assumed to be uniformly distributed between plasma and red blood cells.

### Kinetic Analysis and Model Validation

Three different kinetic models were evaluated in this analysis: a reversible one-tissue compartment model with two rate constants (1T2k), an irreversible two-tissue compartment model with three rate constants (2T3k), and a reversible two-tissue compartment model with four rate constants (2T4k). Additionally, the fractional blood volume component was estimated for each model. Model optimality was evaluated based on chi-square goodness of fit criteria and the Aikake information criterion (AIC), consistent with other studies [8]. The 2T3k model net $influx\;rate=K_{1}k_{3}/\left(k_{2}+k_{3}\right)$ and distribution volume $V_{d}$ were evaluated from the full compartmental model as well as the Patlak graphical method [18].

### Statistical Analysis

Statistical tests were performed with GraphPad Prism 9.5.0 (GraphPad, San Diego, California, USA). Significance was set at 5%, and all variables are reported with median and range or mean and standard deviation. The distributions of all numerical variables were tested for normality. Kinetic and semiquantitative parameters were compared for lesion and reference tissue regions using a patient-wise paired Šídák’s test for multiple comparisons. Linear models and Pearson correlations were calculated to assess the association between compartmental parameters, Patlak graphical parameters, and SUVs. The kinetic models were compared for goodness-of-fit across the central, transitional, and peripheral prostate, and kinetic parameters were compared for consistency across the three prostate zones.

## Results

### Model Selection

**Figure 1:**
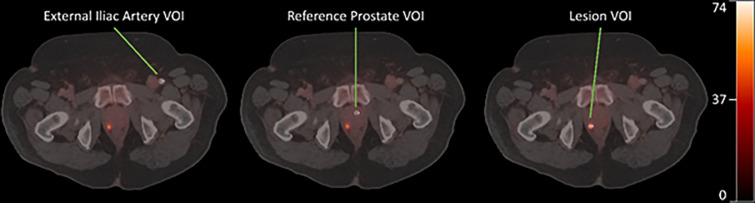
10 mm spherical VOI placements for artery (left), normal prostate tissue (middle), and lesion (right). An early bolus phase image was used to locate the external iliac artery. All three displayed images are 40–55 minute PET frames overlaid with CT. Scale bar is in units of kBq/mL.

**Figure 2:**
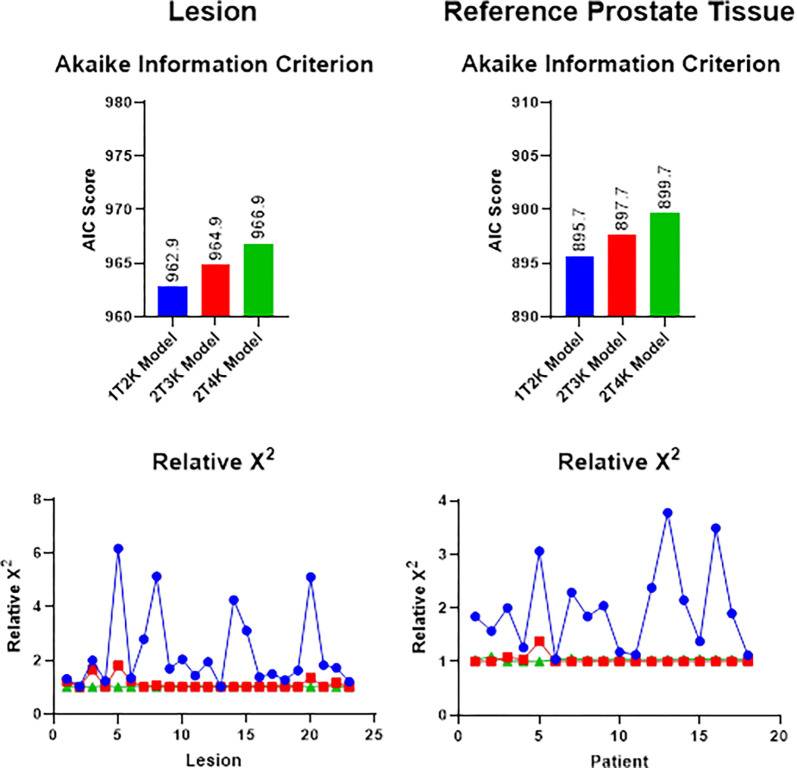
Model selection criteria for three 68Ga-PSMA-11 tracer kinetic models. Blue refers to the one-tissue compartment, two rate constant (1T2K) model. Red shows the two-tissue compartment, three parameter (2T3K) model, and green shows the two-tissue compartment, four parameter (2T4K) model. Relative X2 goodness-of-fit values are normalized to the model with the minimal X2 values for each patient.

An example VOI placement for artery, reference prostate, and lesion is shown in Fig. 1. Model results for the 1T2K, 2T3K, and 2T4K compartmental models are shown in Fig. 2. All three models performed similarly for prostate and reference tissue regions, with a △AIC of 2.0 for the 2T3K model and a △AIC of 4.0 for the 2T4K model, in reference to the 1T2K exchange model. Therefore, the AIC criteria suggests consistent information loss from the 1T2K and 2T3K model, but disfavors the use of the 2T4K model for ^68^Ga-PSMA-11 according to rules established by Burnham and Anderson [19]. Relative $\{\backslash rm\;X{\}}^{2}$ goodness-of-fit criteria support the use of the 2T3K and 2T4K models, as $\{\backslash rm\;X{\}}^{2}$ is significantly reduced for the 2T3K $\left(\{\backslash rm\;X{\}}_{diff}^{2}=14.76,\;df_{diff}=1,\;p<0.001\right)$ and 2T4K $\left(\{\backslash rm\;X{\}}_{diff}^{2}=14.43,\;df_{diff}=1,\;p<0.001\right)$ models relative to the 1T2K model, but not relative to each other $\left(\{\backslash rm\;X{\}}_{diff}^{2}=0.33,df_{diff}=1,\;p=0.564\right)$. Therefore, the combination of AIC and $\{\backslash rmX{\}}^{2}$ goodness-of-fit criteria favor the use of the 2T3K model for ^68^Ga-PSMA-11.

### Parametric Evaluation

**Figure 3:**
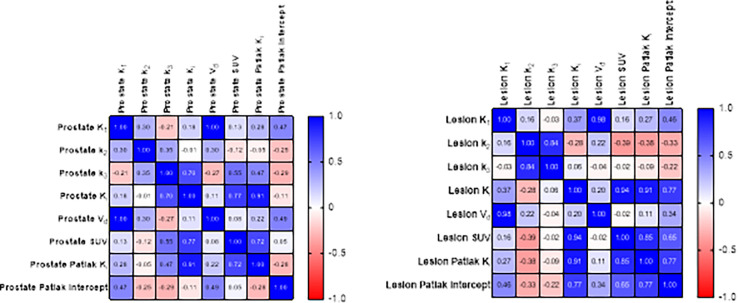
Pearson parameter correlation matrices for reference prostate (N=18) and lesion (N=23) volumes of interest.

**Figure 4:**
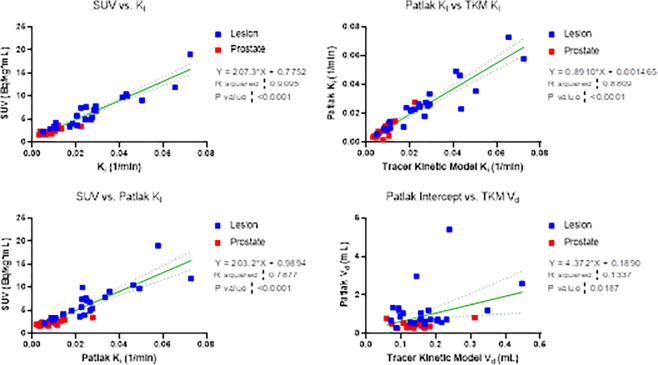
Linear relationships between kinetic parameters, including both reference prostate and lesion volumes of interest. Linear regression results (equation shown) are displayed as a solid green line, with 95% confidence bands in dashed green. P-values indicate a test of significant non-zero regression slope.

**Figure 5:**
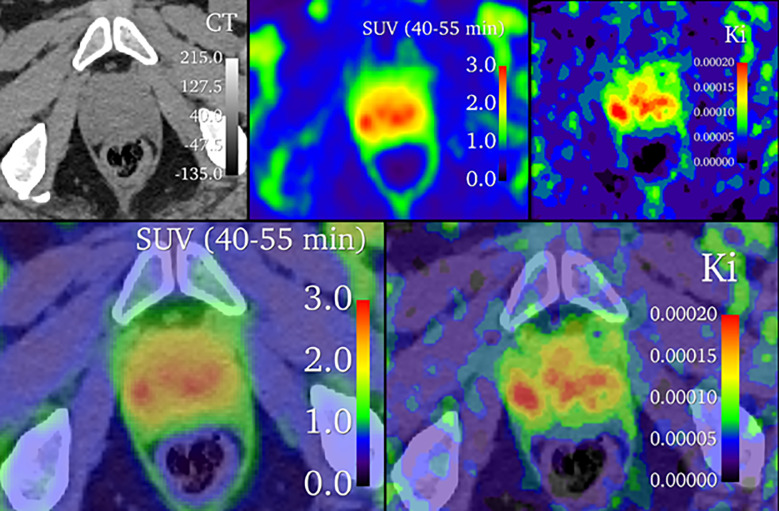
Comparison of SUV and Patlak K_i_^68^Ga-PSMA-11 images. The top row shows parametric images alone, and the bottom row is overlaid with CT at 50% opacity.

An assessment of lesion and reference prostate parameter correlations is shown in Fig. 3. Strong correlations were observed between $K_{1}$ and $V_{d}$ for reference prostate tissue (1.00) and in identified lesions (0.98). Additionally, the net influx rate, $K_{i}$, demonstrated a strong correlation with SUV in reference prostate and lesions, whether it was calculated by full compartmental analysis or Patlak graphical analysis. Accordingly, the Pearson correlation between the compartmental model $K_{i}$ and Patlak graphical model $K_{i}$ was 0.91. However, there was a weak positive correlation between the full compartmental model $V_{d}$ and the Patlak model intercept terms, especially within lesions. Linear regressions between SUV, $K_{i}$, Patlak $K_{i},\;V_{d}$, and Patlak intercept are shown in Fig. 4 and Supplemental Fig. 1, demonstrating large coefficients of determination between SUV, $K_{i}$, and Patlak $K_{i}$. However, differential uptake patterns can be observed between SUV and Patlak $K_{i}$ images, as shown in Fig. 5.

**Figure 6:**
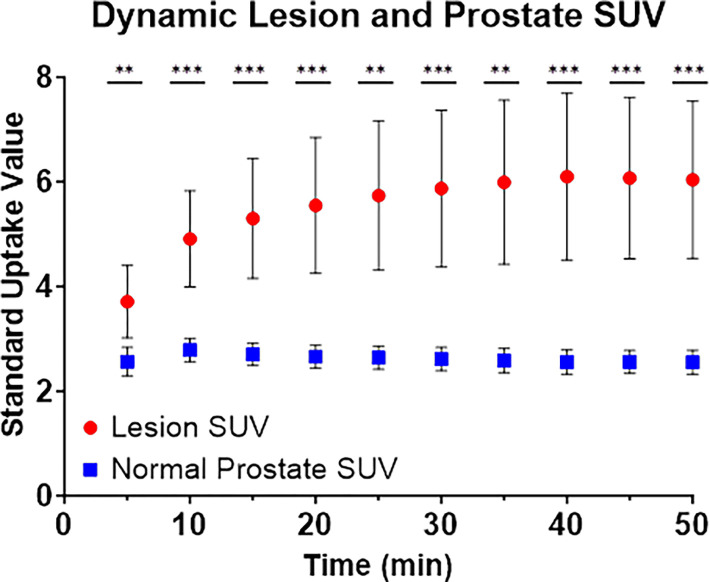
Comparison of standard uptake value (SUV) temporal trends in normal prostate and lesion. Shown is the mean SUV for prostate and lesion at 5 minute intervals, calculated as the uptake over a sliding 10-minute window centered over each timepoint. Error bars represent 95% confidence intervals of the mean. Statistical tests convey the results of a patient-wise paired T-test at each timepoint. p<0.01 is indicated by **, and p<0.001 is indicated by ***.

Median, first, and third quartile parameter values are charted for reference prostate tissue and lesions in Table 2. Matched lesion and reference prostate parameter values are included in Supplemental Fig. 2. Significant differences between parameter values for lesion and reference prostate are noted for $k_{1},\;k_{2},\;k_{3},\;K_{i}$, and SUV in Table 3. Additionally, temporal variations in normal prostate and lesion SUV are compared in Fig. 6. Despite parametric differences between lesion and reference prostate, no significant differences in zone-specific kinetic rate constants or model goodness-of-fit were observed (Supplemental Fig. 3 and Supplemental Fig. 4).

## Discussion

The results of this study support a two-tissue, three-parameter kinetic model for characterizing the pharmacokinetics of the ^68^Ga-PSMA-11 radiopharmaceutical. ^68^Ga-PSMA-11 exhibits free binding to the extracellular domain of PSMA and slow cellular internalization[20, 21], thus providing a physiological basis for the irreversible two-tissue compartment model and Patlak analysis. In comparison with other primary evaluations of ^68^Ga-PSMA-11 kinetics, the median PSA of patients reported in this study is reduced (6.8ng/mL) versus Sachpekidis et al. (24.1ng/mL) and Ringheim et al (8.64ng/mL). Additionally, 11/18 patients possessed favorable intermediate grade disease, in comparison with the greater proportion of high risk disease in other kinetics studies [14, 22].

Although the Akaike information criterion suggested that maximal information was preserved by the 1T2k model, chi-square goodness-of-fit criteria suggested that the 1T2k model did not appropriately fit ^68^Ga-PSMA-11 time-activity curves. Therefore, the 2T3k model is optimal based on dual consideration of the Akaike information criterion and chi-square goodness-of-fit criteria. Previous kinetic evaluations of ^68^Ga-PSMA-11 for primary prostate cancer have supported the 2T3k or the 2T4k kinetic models, but the findings of this study are consistent with the analysis in high-risk patients established by Ringheim, et al. [13, 14, 22].

Kinetic parameters $\left(K_{1},k_{2},k_{3},K_{i}\right)$ exhibited significant differences between lesion and reference prostate tissue, as demonstrated by patient-wise comparison (Fig. 4, Supplemental Fig. 2) and statistical comparisons (Table 3). The sampled compartmental model rate constants were also consistent with those reported by Ringheim, et al.[14]. Parameter differences between lesion and reference prostate remained significant, regardless of whether $K_{i}$ estimates were obtained by full compartmental models or Patlak graphical analysis. $K_{i}$ values obtained through compartmental modeling and Patlak analysis were substantially correlated for reference prostate tissue and lesions (Pearson r=0.91).

The current EANM/SNMMI ^68^Ga-PSMA-11 image acquisition guidelines recommend an acquisition timeframe between 50 and 100 minutes post-injection, primarily due to increasing time-activity curves and SUVs in late time-windows relative to normal prostate [23–26]. However, it has also been reported that tumor visibility is improved in the 30–45 minute window, where bladder accumulation is reduced and statistical count rates remain high[14, 27]. Here, we find that kinetic and semiquantitative parameters can discriminate lesion from reference prostate using earlier acquisition protocols, even as early as 30 minutes post-injection as shown by Fig. 6. Additionally, although previous reports have also favored the lean body mass SUV over body-weight SUV, we show a strong concordance (r^2^ = 0.9281, Supplemental Fig. 1) between the percentage of the injected dose per gram and body-weight SUV semiquantitative analysis metrics[28].

Consistent with other reports, lesion $K_{i}$ values correlated strongly with SUV for compartmental (Pearson r = 0.94) and Patlak (Pearson r=0.85) models, indicating that 40–55 minute post-injection SUV metrics provide substantially similar information as $K_{i}$ values[14, 22]. Maximal SUVs have also been found to correlate with immunohistochemical PSMA expression and histopathology in patients with prostate cancer [29, 30]. Therefore, it is unlikely that $K_{i}$ provides additional information beyond that of either the percentage of the injected dose per gram or the measured SUV, a simpler method which is already commonplace in many clinical workflows. Instead, $K_{i}$-based images may have utility in imaging of cancers with lower levels of PSMA expression, where improvements in lesion-to-normal tissue contrast from blood pool signal reduction may be more impactful towards differentiating lesions from the image background.

Although $K_{i}$ (Patlak and full compartmental analysis) correlated strongly with SUV, individual compartment rate constants ($K_{1},\;k_{2},\;k_{3}$) demonstrated minimal to slightly negative correlation with SUV. Table 2 and Table 3 demonstrate that $K_{1}$ and $k_{3}$ are significantly elevated in lesions, while $k_{2}$ is significantly decreased. These findings are consistent with increased PSMA expression and PSMA-11 internalization on the prostatic epithelium. In contrast to $K_{i}$, the low correlation of kinetic parameters with SUV indicates that they provide additional independent information of tissue physiology. Therefore, kinetic analysis with the Patlak method is not expected to provide additional diagnostic utility, and the 2T3k compartmental model is the preferred method of dynamic analysis for ^68^Ga-PSMA-11 PET.

While $K_{i}$ and $V_{d}$ were uncorrelated parameters in the 2T3k compartmental model (Pearson r ≤ 0.2 for reference prostate and lesion), Patlak $K_{i}$ and $V_{d}$ were correlated (Pearson r=0.77) in lesions and moderately anti-correlated (Pearson r=-0.28) in prostate reference tissue. Through a preliminary investigation, the Patlak $K_{i}∙V_{d}$ product was at least three times the value in lesions when compared to reference prostate tissue for all 18 patients. Further investigation is necessary to determine if parameter combinations are diagnostically relevant.

As noted in Supplemental Fig. 3 and Supplemental Fig. 4, there were no statistically significant differences between compartmental rate constants, compartmental model $K_{i}$, or $V_{d}$ between normal prostatic tissue in the central, transitional, and peripheral prostatic zone. Additionally, the chi-square goodness-of-fit criterion was consistent across all prostatic zones, indicating that the model is likely appropriate regardless of prostatic location. This observation is in contrast to previously reported findings by Pizzuto, et al, who reported that ^68^Ga-PSMA-11 accumulation is higher in the central zone than in the transition or peripheral zone [31]. However, the finding was reported during the staging for high-risk disease, and thus could be attributable as a feature of aggressive disease. In our study, 11% (2/18) patients met or exceeded the average SUVmean reported by Pizzuto, et al. in the central zone.

This study, although consistent with other literature reports in its findings, has several limitations which should inform its interpretation. First, the small sample size limits statistical power and result generalizability. Additionally, the scope of patients included in this study is primarily limited to intermediate-risk disease, as only patients who were candidates for prostatectomy received ^68^Ga-PSMA-11 PET/CT scans. The study included no low-risk patients, and only a single high-risk patient. Additionally, the demographics of patients meeting the study risk criteria were highly racially homogeneous. The present study was performed on presurgical research scans, and did not acquire list-mode data past 55 minutes. Thus, the kinetics and late time-frame SUV images are temporally constrained and do not fully utilize the timeframes recommended by EANM and SNMMI[23].

## Conclusion

The two-tissue irreversible compartment model is appropriate for kinetic analysis in ^68^Ga-PSMA-11 imaging. The two-tissue irreversible compartment model is applicable to central, transitional, and peripheral prostate, regardless of tumor involvement. Kinetic parameters ($K_{1},\;k_{2},\;k_{3}$) are useful towards distinguishing prostate cancer lesions from normal prostatic tissue, and kinetic parameters provide information about tissue physiology that is independent from SUV-based metrics and Patlak ($K_{i}$) net influx rate.

## Figures and Tables

**Table 1.** Patient characteristics, injected doses, and summary pathology classification.

Subject	Age	Prostate Volume (cc)	PSA (ng/mL)	Subject Weight (kg)	Injected Dose (MBq)	Number of Lesions	Lesion Location (Gleason Grade Group)
1	69	35.2	9.6	108.4	183.2	1	RTZ (2), RCZ (1)
2	62	36.1	20.6	132.0	180.6	1	LPZ (3)
3	69	60	9.8	76.2	183.5	1	RCZ (4), RPZ (3)
4	69	39.1	5.6	97.5	183.2	1	LPZ (3)
5	63	30	4.2	79.4	181.3	1	LPZ (2)
6	57	28.5	5.6	90.7	170.6	1	RPZ (2)
7	52	27.3	6.2	111.1	171.7	2	RTZ (2), RPZ (2), LTZ (2)
8	57	51	10.8	108.0	182.4	2	LCZ (2), LTZ (2), RPZ (2)
9	64	43.8	4.8	83.9	185.0	1	RPZ (3)
10	66	59.4	18.5	88.5	185.0	1	LPZ (3)
11	53	37.9	9.2	91.2	183.9	1	LCZ (3)
12	69	66.5	8.7	63.5	184.6	1	LPZ (3)
13	60	41.6	4.5	92.1	186.1	1	RPZ (2)
14	75	37.4	6.6	81.7	184.6	1	LPZ (2)
15	58	62.8	6.7	112.0	185.7	1	RPZ (2)
16	66	41.9	6.8	90.7	183.2	2	LCZ (2), RPZ (1)
17	66	89.4	7.3	69.0	184.3	2	RTZ (2), RPZ (2)
18	67	72.6	4.1	88.0	183.5	2	RPZ (2)

PSA - prostate specific antigen. The lesion locations are described as the anatomical left (L) or right (R) central zone (CZ), transitional zone (TZ), or peripheral zone (PZ) based on postsurgical whole-mount pathology.

**Table 2.** Median parameter values from 23 lesions in 18 patients. Shown are comparative median, mean, standard deviation, first, and third quartile parameter values in lesions and reference prostate tissue.

Parameter	Lesion *(n=23)*	Prostate *(n = 18)*
SUV	5.73 [6.74, 3.83, 3.84–8.47]	2.33 [2.36, 0.59, 1.91–2.65]
%ID/kg	6.64% [7.21 %, 3.47%, 4.77% – 8.74%]	2.59% [2.58%, 0.5%, 2.35% – 2.91%]
K_1_(mL/min/mL)	0.192 [0.199, 0.094, 0.141–0.222]	0.145 [0.147, 0.055, 0.121–0.168]
k_2_ (/min)	0.182 [0.247, 0.245, 0.110–0.292]	0.317 [0.317, 0.094, 0.254–0.369]
k_3_ (/min)	0.032 [0.035, 0.029, 0.019–0.038]	0.020 [0.020, 0.010, 0.011–0.026]
K_i_ (/min)	0.025 [0.028, 0.018, 0.018–0.035]	0.008 [0.008, 0.005, 0.005–0.010]
V_d_ (L)	0.157 [0.171, 0.089, 0.101–0.204]	0.136 [0.138, 0.054, 0.116–0.156]
Patlak K_i_ (mL/min/mL)	0.024 [0.027, 0.017, 0.016–0.030]	0.008 [0.009, 0.006, 0.005–0.011]

Shown are median [mean, standard deviation, first quartile - third quartile] value comparisons for lesion and normal prostate. Semiquantitative values include the standard uptake value (SUV) and percent injected dose per gram (%ID/g). Quantitative parameters include the kinetic two-tissue, three rate constant model parameters, net influx rate (*K_i_*), distribution volume (*V_d_*), and Patlak model net influx rate (Patlak *K_i_*).

**Table 3.** Comparison of semiquantitative and quantitative parameter values between reference prostate and lesion.

Šidák’s multiple comparisons test	Summary	Adjusted P Value
Prostate K_1_ vs. Lesion K_1_	**	0.0080
Prostate k_2_ vs. Lesion k_2_	**	0.0084
Prostate k_3_ vs. Lesion k_3_	**	0.0080
Prostate K_i_ vs. Lesion K_i_	****	<0.0001
Prostate V_d_ vs. Lesion V_d_	ns	0.6425
Prostate SUV vs. Lesion SUV	****	<0.0001
Prostate Patlak K_i_ vs. Lesion Patlak K_i_	****	<0.0001
Prostate Patlak Intercept vs. Lesion Patlak Intercept	****	<0.0001
Prostate K_i_ vs. Prostate Patlak K_i_	ns	>0.9999
Prostate V_d_ vs. Prostate Patlak Intercept	****	<0.0001

Significance level is set at 0.05, and is corrected for multiple comparisons with the Šidák correction.

## Data Availability

*Data for this publication were acquired in conjunction with* NCT04936334. All data and materials presented in this manuscript will be made available upon reasonable request to the corresponding author.

## Abbreviations

PET: positron emission tomography
PSMA: prostate-specific membrane antigen
^68^Ga: PSMA-11 ^68^Ga-Glu-NH-CO-Lys-(Ahx)-HBED-CC
SUV: standard uptake value
NCCN: National Comprehensive Cancer Network
OSEM: ordered-subsets expectation maximization
PSF: point-spread function
TOF: time of flight
CT: computed tomography
ROI: region of interest
TAC: time-activity curve
IFIF: image-derived input function
1T2K: one-tissue, two-parameter
2T3K: two-tissue, three-parameter
2T4K: two-tissue, four parameter
AIC: Akaike information criterion
EANM/SNMMI: European Association of Nuclear Medicine/Society of Nuclear Medicine and Molecular Imaging
